# Neonatal Hypopituitarism: Approaches to Diagnosis and Treatment

**DOI:** 10.4274/jcrpe.galenos.2018.2018.0036

**Published:** 2019-02-20

**Authors:** Selim Kurtoğlu, Ahmet Özdemir, Nihal Hatipoğlu

**Affiliations:** 1 Erciyes University Faculty of Medicine, Department of Pediatrics, Division of Neonatalogy, Kayseri, Turkey; 2 Erciyes University Faculty of Medicine, Department of Pediatrics, Division of Pediatric Endocrinology, Kayseri, Turkey

**Keywords:** Diagnosis, hypophysis, hypothalamus, neonatal hypopituitarism, treatment

## Abstract

Hypopituitarism is defined as a decreased release of hypophyseal hormones, which may be caused by disease of the pituitary gland disease or hypothalamus. The clinical findings of neonatal hypopituitarism depend on the causes and on presence and extent of hormonal deficiency. Patients may be asymptomatic or may demonstrate non-specific symptoms, but may still be at risk for development of pituitary hormone deficiency over time. Patient history, physical examination, endocrinological, radiological and genetic evaluations are all important for early diagnosis and treatment. The aim of this paper was to present a review of etiological factors, clinical findings, diagnosis and treatment approaches in neonatal hypopituitarism.

## Introduction

The pituitary gland is the central regulator of growth, metabolism, reproduction and homeostasis. It consists of a frontal lobe (adenohypophysis), a posterior lobe (neurohypophysis) and a middle lobe. Growth hormone (GH), follicle stimulating hormone (FSH), luteinising hormone (LH), thyroid stimulating hormone (TSH), prolactin (PRL) and adrenocorticotropic hormone (ACTH) are released from the frontal lobe and arginine vasopressin (AVP) and oxytocin from the posterior lobe. The frontal and middle lobes consist of ectoderm and the posterior lobe consists of neural ectoderm (1). A series of transcription factors are involved in pituitary gland formation. Neonatal hypopituitarism may occur due to developmental defects of the pituitary gland, genetic mutations, and perinatal and neonatal events (Table 1) (1,2,3,4,5,6,7,8). Genetic mutations causing hypopituitarism and their sub-groups are shown in Tables 2 and 3 (1,9,10,11,12,13,14). The incidence of congenital hypopituitarism is estimated to be between 1/4000-1/10,000 (15).

Hypopituitarism findings may not be present in the neonatal period and may occur with different, non-specific, clinical presentations. Also, the sensitivity of laboratory methods may not be satisfactory for newborns (13). Nevertheless, it is possible for neonatologists to reach a diagnosis by focusing on some clues.

## Neonatal Clinical Findings in Congenital Hypopituitarism Cases

**Figure 1:**
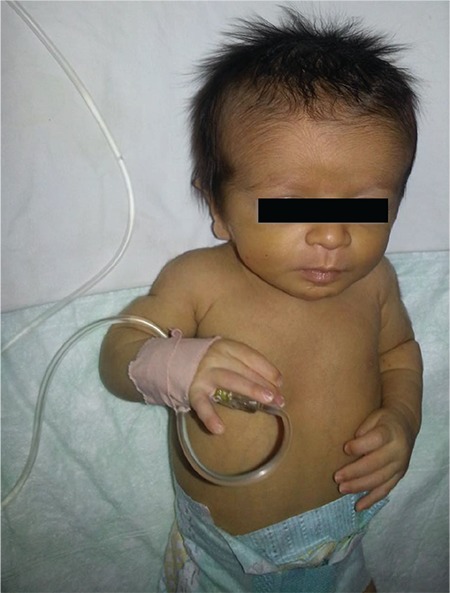
Facial appearance in the presence of neonatal growth hormone deficiency (from the files of Erciyes University Faculty of Medicine, Department of Neonatology)

**Figure 2:**
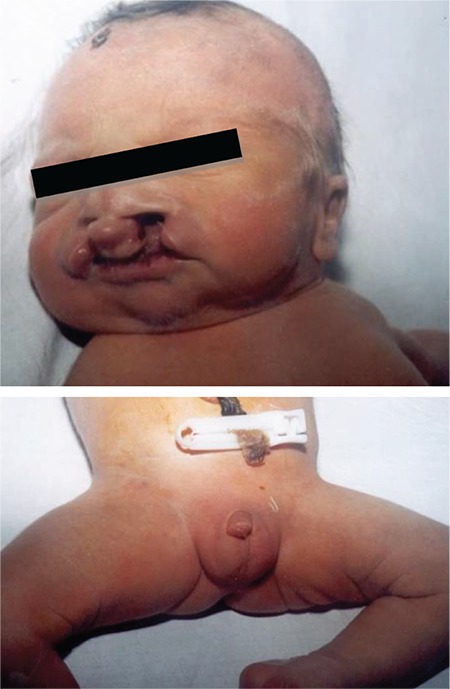
Midline defect with cleft palate-lip and micropenis (from the files of Erciyes University Faculty of Medicine, Department of Neonatology)

**Figure 3:**
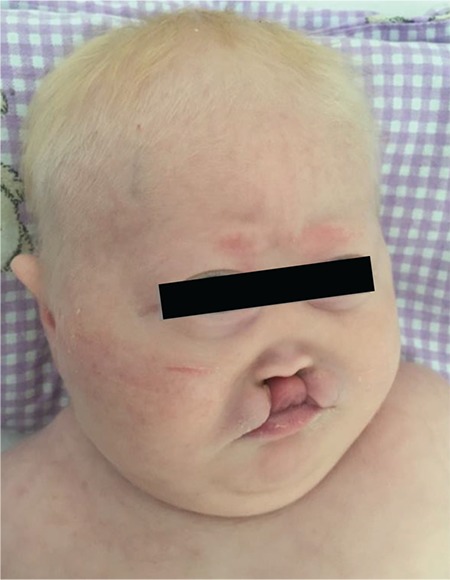
A case of holoprosencephaly with cleft palate/lip and anterior and posterior pituitary insufficiency (from the files of Erciyes University Faculty of Medicine, Department of Neonatology)

It is interesting that newborns with congenital hypopituitarism have normal birth weight and height (16). Clinical presentations in hypopituitary newborns occur due to combined or total hypophyseal hormone deficiency. Ocular findings, midline defects and genital abnormalities may also be detected in these patients (Figures 1, 2, 3).

Generally these patients present with non-specific findings although clinical findings may become evident over time. All newborns suspected of hypopituitarism must be assessed for optic nerve hypoplasia, midline defects or syndromes; even those in whom the initial endocrine evaluations are normal (17,18,19). In premature infants diagnosis is difficult due to problems commonly associated with prematurity including hypothalamus-pituitary axis immaturity and limited information on normal values for newborns and contraindication of stimulation tests. It is reported that only 23% of cases are diagnosed with postnatal problems such as hypoglycemia, hyponatremia or recurrent sepsis in the neonatal period (20). Non-specific symptoms such as hypoglycemia, neuroglycopenia-related lethargy, apnea, jitteriness, convulsions, inability to gain weight, hyponatremia unaccompanied by hyperkalemia, temperature instability, recurrent sepsis, hemodynamic instability, neonatal cholestasis and prolonged jaundice are observed in the neonatal period (21,22,23). In addition to hypoglycemia, lack of thymic involution and fluid intolerance are striking in cortisol deficiency (24). Cortisol deficiency-related hypoglycaemia, as a result of isolated or combined-type ACTH deficiency, is severe. Heart failure constitutes a vital risk in newborns with the *LHX4* mutation-related multiple hormone deficiency. Heart failure in these newborns can be resolved with thyroxine and hydrocortisone treatment and the hypoglycemia can be treated successfully with GH (25). Cortisol increases bile flow and cortisol deficiency leads to problems in bile acid synthesis and transport and eventually cholestasis. Cholestasis occurs generally in the first 13 days of life. Transaminase levels increase after 2-4 weeks but GGT remains within normal ranges (26). In cholestasis cases, liver biopsy, usually performed before hypopituitarism diagnosis, reveals canalicular cholestasis. Mild portal eosinophilic infiltration is demonstrable on histopathology. If there is a delay in diagnosis, transaminase levels continue to increase, while cholestasis recovers in 6-10 weeks if treatment is started after diagnosis (27,28). ACTH deficiency is present in over 50% of cases with ocular and frontal brain abnormalities. Temperature instability and prolonged physiological jaundice are also usually present in cases with neonatal TSH deficiency. The development of female genitalia is independent of hormone secretion; hence congenital hypogonadotropic hypogonadism (HH) is not expected to affect the normal development of female external genitalia (29). Micropenis is defined according to a -2.5 standard deviation cut-off from the mean value. Values under 1.5 cm at gestational age 30 weeks, 2 cm at 34 weeks and under 2.5 cm in term infants are defined as micropenis (30). Optic nerve hypoplasia or corpus callosum agenesis-related nystagmus may be observed in infants (31,32). Polyhydramniosis, polyuria, weight loss, anxiety, demand for water instead of formula, signs and symptoms of dehydration and hypernatremia are observed in cases of diabetes insipidus (33).

## Diagnostic Approaches in Neonatal Hypopituitarism

**Patient and family history:** A careful and detailed medical history should be obtained including information on consanguineous marriage, index cases, traumatic/breech birth and possible neonatal central nervous system infection.

**Physical examination findings and symptoms:** Height, weight and head circumference should be measured in the newborns. Fontanelle size, eyes, cleft palate/lip, hepato-splenomegaly, lymphadenopathy, jaundice and malformations are assessed. Presence of microphallus and undescended testicles are noted in the genital examination.

Syndromes accompanied by hypophyseal deficiency are listed in Table 4 (1).

## Endocrine Evaluation

**Pituitary-adrenal axis:** ACTH deficiency may be life threatening. Quick action is important, especially with asymptomatic midline defects. Circadian rhythm in cortisol secretion does not mature in the first six postnatal months. Thus, cortisol should be measured every hour of the day instead of only in the morning (34). Mehta et al (21) interpret cortisol values below 175 nmol/L (6.34 micrograms/dL) at 8 o’clock in the morning as deficiency. Multiple random cortisol measurements are not suitable for premature and ill infants and cortisol measurement by induced hypoglycemia is not recommended. However, cortisol measurement may be useful in addition to insulin and GH measurement in infants with hypoglycemia at presentation. Cortisol deficiency is accepted to be present if cortisol response remains below 12.67 micrograms/dL in hypoglycemic infants (35). While a standard ACTH test is easy and safe, the sensitivity is approximately 80% (10). False negative results can occur even in infants with ACTH deficiency (36). A corticotropin releasing hormone test can be performed to determine ACTH deficiency in infants. However, normative values in cases of central hypothyroidism and midline defects are not known and the test is contraindicated in ill infants (37). As the circadian rhythm matures, a cortisol value of 175 nmol/L (6.34 micrograms/dL) at 8 o’clock in the morning excludes ACTH deficiency if the cortisol level is above 540 nmol/L (19.56 microgram/dL) at the 30th minute with a low dose ACTH test. The specificity of the test was found to be 100% but the sensitivity was 69% (20).

**TSH deficiency:** In cases of central hypothyroidism, low or normal TSH level despite a low FT4 level is striking. Central hypothyroidism is diagnosed if the free T4 level is below 0.8 ng/dL and TSH is normal or slightly elevated (38). It should be kept in mind that severe infection, sick thyroid syndrome or dopamine infusion can cause low TSH levels in infants (39). Early diagnosis is important in central hypothyroidism cases since other hormone deficiencies may be concurrent in as high as 78% of cases (1). Hypoglycemia and neonatal hepatitis can develop if hypothyroidism is diagnosed late and mortality is reported to approach 14% in these patients (36). The necessity of a TRH test for diagnosis is controversial (40,41).

**Gonadotropin deficiency:** Micropenis alone or together with undescended testicles in boys is observed in isolated HH or in multiple hypophyseal hormone deficiencies. Secretion of postnatal FSH and LH occurs in normal male newborns and testosterone levels increase with a peak in the 4-10^th^ weeks and start to decrease around the sixth month. High gonadotropin levels may last for up to two years in girls. This series of events is called mini-puberty (42). If LH is <0.8 IU/L and total testosterone is <30 ng/dL in male infants between postnatal day 15 and six months, central hypogonadism is diagnosed (43). In female infants, central hypogonadism is diagnosed if FSH is <1.0 IU/L between day 15 and two years (44,45,46,47). HH can be diagnosed with gonadotropin releasing hormone and human chorionic gonadotropin (hCG) tests in infants (48,49). Basal FSH and LH are low in infants with HH and a blunt gonadotropin response is seen after the test (37). Attention should be paid to penile growth and testicular descent in infants tested with hCG (50).

**GH deficiency:** It should be kept in mind that GH level is high in the neonatal period (51,52). GH can be measured directly in the neonatal period although a decrease in the GH level and an increase in insulin-like growth factor-1 (IGF-1) and IGF binding protein-3 (IGFBP-3) levels are observed in the neonatal period, starting from birth. Kurtoğlu et al (53) reported that in term infants GH levels decreased and IGF-1 and IGFBP-3 levels showed a gradual increase (Table 5). Binder et al (54), reported GH levels in the neonatal period in children who were later detected to have GH deficiency. They concluded that in the postnatal first week, a level of 7 ng/mL reflected GH deficiency and this level showed very good sensitivity and specificity-100% and 98% respectively. The same group also observed that neonatal GH deficiency was present in cases who had multiple hormone deficiency and malformations, but that isolated GH deficiency was not detected in newborns (55). It has also been reported that random GH measurement may be useful in the first 14 days in newborns (56).

Although GH stimulation tests are not recommended in infants under 12 months old, GH levels measured in infants with hypoglycemia may yield useful clinical clues to diagnosis, though the specificity is low (57,58). A GH value of <7.7 ng/mL in infants with hypoglycemia has been suggested as a criterion of GH deficiency (35). It has been reported that a glucagon stimulation test may be used in infants younger than 12 months of age (59). When glucagon is injected at a dose of 0.03 mg/kg and samples are taken at basal, 45, 90, 120, 150 and 180 minutes, the GH level is normally expected to be above 10 ng/mL.

**PRL deficiency:** PRL concentration is low in cases with *POU1F1*, *LHX3*, *OTX2* and *IGFSF-1* gene mutations and in cases of panhypopituitarism. Values below 31 ng/mL in the first 30 postnatal days and 24 ng/mL between the 30^th^-60^th^ days are accepted as hypoprolactinemia (60). Breast tissue should not be palpated before taking blood and it should be confirmed that no medicine affecting PRL level has been taken. PRL levels may be low in infants given dopamine as an inotropic agent in the neonatal period (61).

**Diabetes insipidus:** The definition for polyuria in diabetes insipidus is a daily urine output >2 liters/m^2^, which corresponds to a volume of 150 mL/kg/day in the newborn (62). The suggested criterion for polyuria of 4 mL/kg/h in children corresponds to a urine volume of >6 mL/kg/hour in the neonatal period (63). In most cases of diabetes insipidus presenting in the neonatal period, anatomical defects or autosomal dominant-recessive genetic causes are present. Central diabetes insipidus presentation is also observed in cases with septo-optic dysplasia, corpus callosum agenesis and holoprosencephaly (64). Very rarely, tumours located in the posterior hypophysis and surgical intervention for craniopharyngioma cause diabetes insipidus. Symptoms such as polyuria, polydypsia (excessive drinking of water rather than formula), weight loss, growth deficiency and persistent hypernatremia despite giving fluid and dilute urine may be striking. Plasma and urine osmolarity measurements in the early hours of the morning may help in ascertaining a diagnosis (1). Serum osmolarity <270 mosm/kg and urine osmolarity >600 mosm/kg draw suggest an alternative diagnosis rather than diabetes insipidus. A water deprivation test is risky in the neonatal period and can only be done in special centers (33). However, a nasal desmopressin test may be performed. On the 8^th^ and 24^th ^hours after the application of 0.012 mL of desmopressin (1.2 microgrammes; 1 mL=100 microgram), observation of a decrease in serum sodium and osmolarity, an increase in urine osmolarity and a decrease in diuresis support the diagnosis (65).

**Radiological examinations:** In newborns, bone age is assessed with knee radiography. Epiphyseal dysgenesis should also to be investigated. Brain and hypophysis imaging should be done in infants suspected of having hypopituitarism. The degree of severity of the hypopituitarism will be proportional to the extent of the neuro-radiological abnormalities (18). Pituitary gland height neurohypophysis brightness or ectopia, an undescended posterior lobe, infundibulum morphology, absence of corpus callosum and of septum pellucidum, optic nerve and chiasma, holoprosencephaly, schizencephaly, cerebellar hypoplasia, absence of fornix and presence of Chiari malformation should be assessed with imaging (66). Lack of neurohypophysis brightness supports the diagnosis in cases of central diabetes insipidus. Data on pituitary gland height in newborns are presented in Table 6 (67,68,69,70).

**Genetic studies:** Genetic studies should be targeted depending on family history, physical examination and laboratory and radiological findings (13,15).

## Treatment Approaches and Follow-up

Cases diagnosed with neonatal hypopituitarism should be followed-up by a multidisciplinary team. Suitable hormonal treatments, providing follow-up baring in mind that some hormone deficiencies may develop slowly, ocular and neurodevelopmental follow-up in syndromic cases, acquisition of genetic data and establishing a good relationship with the family are important during follow-up. 

Treatment of central hypothyroidism is started with 6-8 microgram/kg/day L-thyroxine. The aim is to keep the free T4 level in the upper part of the normal range. After starting treatment dose insufficiency is monitored by measuring free T4 and overdose by free T3 concentrations (71). It is important to know the cortisol level before thyroxine replacement. This is because cortisol clearance increases and cortisol deficiency occurs when thyroxine is given to infants with low cortisol level, especially in preterm cases (72). In cases of cortisol deficiency oral hydrocortisone should be started first and thyroid replacement should be initiated subsequently. In preterm infants, daily cortisol production is reported to be 7.28 mg/m^2^/day on the fifth day and 6 mg/m^2^/day in the second week (73). Oral cortisol should be higher than daily production and should be given by dividing into three doses of 12-18 mg/m^2^/day. In case of stress, the dose should be increased two- to threefold. Diabetes insipidus may develop with hydrocortisone treatment and the infant should be observed closely (33). In infants with cholestasis at initiation of treatment oral thyroxine and hydrocortisone should be administered at high dose due to absorption deficiency and it should be kept in mind that the doses should be decreased after cholestasis resolves (74).

Testosterone injection, dihydrotestosterone gel application or recombinant human gonadotropin subcutaneous infusion treatments can be initiated between the ages of 1-6 months in boys in whom HH is diagnosed (30,75,76). Acceptable results were obtained by giving 25 mg depot testosterone intramuscularly, every three weeks over a period of three months (77).

Intranasal or peroral desmopressin should be used in cases of central diabetes insipidus. The maximum plasma concentration was observed after 40-55 minutes with intranasal or oral use and the half-life is nearly 3.5 hours. Urine output starts to decrease after 1-2 hours and the effect lasts from six to 18 hours (78). It is recommended to start treatment with a low dose and titrate according to the response. The intranasal form should be started with a dose of 0.05-0.1 micrograms and should also be titrated (79). Oral tablets are dissolved in 3-5 mL of water and given by dividing the daily dose of 5 micrograms/kg into two (80,81). When the treatment is started, the daily liquid intake should be lowered to maintain fluid quantities which will prevent hyponatremia (65,80).

GH treatment can be started in the neonatal period. However. treatment is often started after the neonatal period since diagnosis is generally delayed. GH treatment can contribute to hypoglycemia recovery (16).

## Conclusion

Neonatal period is different from other periods of life. Assessment of hypothalamus-hypophysis axis different from the other stages of life. The hormonal deficiencies particularly in this period being asymptomatic make the interpretation of pathologic conditions difficult. Therefore, efforts have been made to shed light on the diagnosis and the therapeutic approach specific to this period.

## Figures and Tables

**Table 1:**
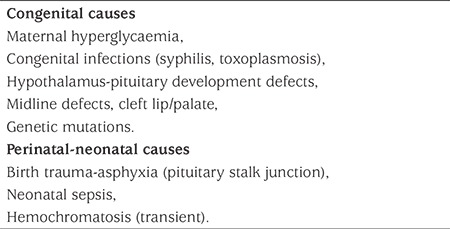
Causes of neonatal hypopituitarism

**Table 2:**
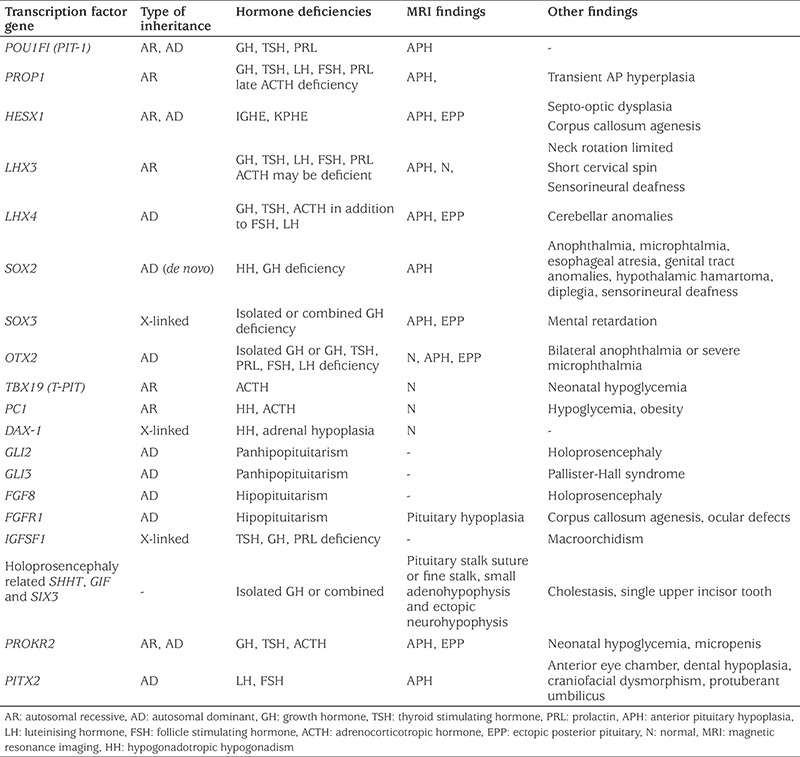
Mutations and characteristics of genes involved in pituitary gland development

**Table 3:**
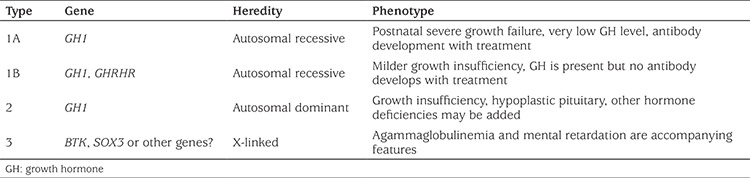
Isolated growth hormone deficiency subtypes

**Table 4:**
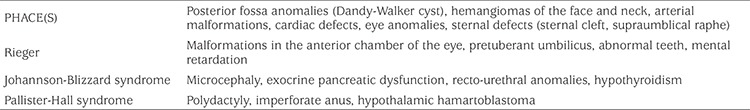
Some syndromes with pituitary insufficiency

**Table 5:**
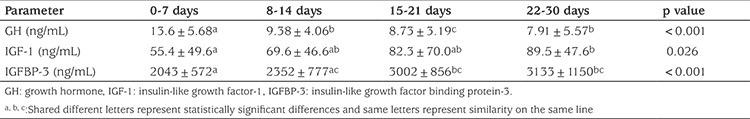
Weekly growth hormone, insulin-like growth factor-1 and insulin-like growth factor binding protein-3 values in the neonatal period (given as means ± standard deviation). The p value shows significant change over time

**Table 6:**
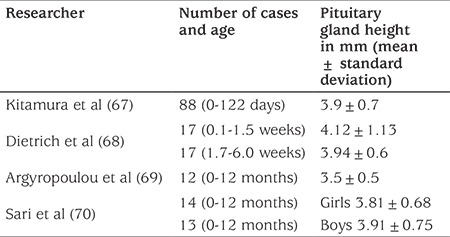
Pituitary gland height values in the neonatal period (mm)

## References

[1] Alatzoglou KS, Dattani MT (2009). Genetic forms of hypopituitarism and their manifestation in the neonatal period. Early Hum Dev.

[2] Massa G, Vanderschueren-Lodeweyckx M, Van Vliet G, Craen M, deZegher F, Eggermont E (1989). Hypothalamo-pituitary dysfunction in congenital toxoplasmosis. Eur J Pediatr.

[3] Daaboul JJ, Kartchner W, Jones KL (1993). Neonatal hypoglycemia caused by hypopituitarism in infants with congenital syphilis. J Pediatr.

[4] Chang AK, Sopher AB, Pat Gallagher M, Khandji AG, Oberfield SE (2011). Congenital pituitary gland abnormalities--a possible association with maternal hyperglycemia: two case reports. Clin Pediatr (Phila).

[5] Indolfi G, Bèrczes R, Pelliccioli I, Bosisio M, Agostinis C, Resti M, Zambelli M, Lucianetti A, Colledan M, D’Antiga L (2014). Neonatal haemochromatosis with revesible pituitary involvement. Transpl Int.

[6] Saranac L, Bjelakovic B, Djordjevic D, Novak M, Stankovic T (2012). Hypopituitarism occurring in neonatal sepsis. J Pediatr Endocrinol Metab.

[7] Akin MA, Kurtoğlu S, Sarici D, Akin L, Hatipoğlu N, Korkmaz L, Güneş T, Oztürk MA, Akçakuş M (2014). Endocrine abnormalities of patients with cleft lip and/or cleft palate during neonatal period. Turk J Med Sci.

[8] Geffner ME (2002). Hypopituitarism in childhood. Cancer Control.

[9] Bar C, Zadro C, Diene G, Oliver I, Pienkowski C, Jouret B, Cartault A, Ajaltouni Z, Salles JP, Sevely A, Tauber M, Edouard T (2015). Pituitary stalk interruption syndrome from infancy to adulthood: Clinical, hormonal, and radiological assessment according to the initial presentation. PLoS One.

[10] Voutetakis A, Sertedaki A, Dacou-Voutetakis C (2016). Pituitary stalk interruption syndrome: cause, clinical manifestations, diagnosis, and management. Curr Opin Pediatr.

[11] Castinetti F, Reynaud R, Saveanu A, Jullien N, Quentien MH, Rochette C, Barlier A, Enjalbert A, Brue T (2016). MECHANISMS IN ENDOCRINOLOGY: An update in the genetic aetiologies of combined pituitary hormone deficiency. Eur J Endocrinol.

[12] Kelberman D, Dattani MT (2007). Hypopituitarism oddities: congenital causes. Horm Res.

[13] Kelberman D, Dattani MT (2007). Hypothalamic and pituitary development: novelinsights into the aetiology. Eur J Endocrinol.

[14] Arslan D, Patiroğlu T, Kendirci M, Kurtoğlu S (1998). X-linked agammaglobulinemia and isolated growth hormone deficiency. Turk J Pediatr.

[15] Davis SW, Castinetti F, Carvalho LR, Ellsworth BS, Potok MA, Lyons RH, Brinkmeier ML, Raetzman LT, Carninci P, Mortensen AH, Hayashizaki Y, Arnhold IJ, Mendonça BB, Brue T, Camper SA (2010). Molecular mechanisms of pituitary organogenesis: In search of novel regulatory genes. Mol Cell Endocrinol.

[16] Cavarzere P, Biban P, Gaudino R, Perlini S, Sartore L, Chini L, Silvagni D, Antoniazzi F (2014). Diagnostic pitfalls in the assessment of congenital hypopituitarism. J Endocrinol Invest.

[17] Mehta A, Hindmarsh PC, Stanhope RG, Turton JP, Cole TJ, Preece MA, Dattani MT (2005). The role of growth hormone in determining birth size and early postnatal growth, using congenital growth hormone deficiency (GHD) as a model. Clin Endocrinol(Oxf).

[18] Arrigo T, Wasniewska M, De Luca F, Valenzise M, Lombardo F, Vivenza D, Vaccaro T, Coradi E, Biason-Lauber A (2006). Congenital adenohypophysis aplasia: clinical features and analysis of the transcriptional factors for embryonic pituitary development. J Endocrinol Invest.

[19] Ahmad T, Borchert M, Geffner M (2008). Optic nerve hypoplasia and hypopituitarism. Pediatr Endocrinol Rev.

[20] Mehta A, Hindmarsh PC, Mehta H, Turton JP, Russell-Eggitt I, Taylor D, Chong WK, Dattani MT (2009). Congenital hypopituitarism: clinical, molecular and neuroradiological correlates. Clin Endocrinol (Oxf).

[21] Mehta A, Hindmarsh PC, Dattani MT (2005). An update on the biochemical diagnosis of congenital ACTH insufficiency. Clin Endocrinol (Oxf).

[22] Gonc EN, Kandemir N, Sen Y, Yordam N (2005). Hyponatremia can be a presenting finding of multiple pituitary hormone deficiency in children: report of a case and review of literature. Clin Pediatr.

[23] Gönç EN, Kandemir N, Andiran N, Ozön A, Yordam N (2006). Cholestatic hepatitis as a result of severe cortisol deficiency in early infancy: report of two cases and review of literature. Turk J Pediatr.

[24] McQueen MC, Copeland KC (1989). Congenital hypopituitarism. With free water intolerance and lack of thymic involution. Early recognition of clinical presentation. Clin Pediatr (Phila).

[25] Filges I, Bischof-Renner A, Röthlisberger B, Potthoff C, Glanzmann R, Günthard J, Schneider J, Huber AR, Zumsteg U, Miny P, Szinnai G (2012). Panhypopituitarism presenting as life-threatening heart failure caused by an inherited microdeletionin 1q25 including LHX4. Pediatrics.

[26] Sheehan AG, Martin SR, Stephure D, Scott RB (1992). Neonatal cholestasis, hypoglycemia, and congenital hypopituitarism. J Pediatr Gastroenterol Nutr.

[27] Binder G, Martin DD, Kanther I, Schwarze CP, Ranke MB (2007). The course of neonatal cholestasis in congenital combined pituitary hormone deficiency. J Pediatr Endocrinol Metab.

[28] Karnsakul W, Sawathiparnich P, Nimkarn S, Likitmaskul S, Santiprabhob J, Aanpreung P (2007). Anterior pituitary hormone effects on hepatic functions in infants with congenital hypopituitarism. Ann Hepatol.

[29] Braslavsky D, Grinspon RP, Ballerini MG, Bedecarra’s P, Loreti N, Bastida G, Ropelato MG, Keselman A, Campo S, Rey RA, Bergada I (2015). Hypogonadotropic Hypogonadism in infants with Congenital Hypopituitarism: A Challenge to diagnose at an Early Stage. Horm Res Paediatr.

[30] Hatipoglu N, Kurtoglu S (2013). Micropenis: etiology, diagnosis and treatment approaches. J Clin Res Pediatr Endocrinol.

[31] Herman TE, Siegel MJ (2008). Congenital adenohypophysis aplasia. J Perinatol.

[32] Scommegna S, Galeazzi D, Picone S, Farinelli E, Agostino R, Bozzao A, Boscherini B, Cianfarani S (2004). Neonatal identification of pituitary aplasia: a life-saving diagnosis. Review of five cases. Horm Res.

[33] Olilvy-Suart A, Midgley P (2006). Practical Neonatal Endocrinology. Cambridge: Cambridge University Press.

[34] de Weerth C, Zijl RH, Buitelaar JK (2003). Development of cortisol circadian rhythm in infancy. Early Hum Dev.

[35] Padidela R, Kapoor RR, Moyo Y, Gilbert C, Flanagan SE, Ellard S, Hussain K (2010). Focal congenital hyperinsulinism in a patient with septooptic dysplasia. Nat Rev Endocrinol.

[36] Karlsson R, Kallio J, Irjala K, Ekblad S, Toppari J, Kero P (2000). Adrenocorticotropin and corticotropin-releasing hormone tests in preterm infants. J Clin Endocrinol Metab.

[37] van Tijn DA, de Vijlder JJ, Vulsma T (2008). Role of corticotropin-releasing hormone testing in assessment of hypothalamic-pituitary-adrenal axis function in infants with congenital central hypothyroidism. J Clin Endocrinol Metab.

[38] Braslavsky D, Keselman A, Galoppo M, Lezama C, Chiesa A, Galoppo C, Bergadá I (2011). Neonatal cholestasis in congenital pituitary hormone deficiency and isolated hypocortisolism: characterization of liver dysfunction and follow-up. Arq Bras Endocrinol Metabol.

[39] Filippi L, Pezzati M, Cecchi A, Serafini L, Poggi C, Dani C, Tronchin M, Seminara S (2006). Dopamine infusion and anterior pituitary gland function in very low birth weight infants. Biol Neonate.

[40] van Tijn DA, de Vijlder JJ, Verbeeten B Jr, Verkerk PH, Vulsma T (2005). Neonatal detection of congenital hypothyroidism of central origin. J Clin Endocrinol Metab.

[41] Mehta A, Hindmarsh PC, Stanhope RG, Brain CE, Preece MA, Dattani MT (2003). Is the thyrotropin-releasing hormone test necessary in the diagnosis of central hypothyroidism in children. J Clin Endocrinol Metab.

[42] Kurtoğlu S, Baştuğ O (2014). Mini puberty and its interpretation. Turk Pediatri Ars.

[43] Grinspon RP, Bedecarrás P, Ballerini MG, Iñiguez G, Rocha A, Mantovani Rodrigues Resende EA, Brito VN, Milani C, Figueroa Gacitúa V, Chiesa A, Keselman A, Gottlieb S, Borges MF, Ropelato MG, Picard JY, Codner E, Rey RA;, LAREP Group (2011). Early onset of primary hypogonadism revealed by serum anti-Müllerian hormonedetermination during infancy and childhood in trisomy 21. Int J Androl.

[44] Bergadá I, Milani C, Bedecarrás P, Andreone L, Ropelato MG, Gottlieb S, Bergadá C, Campo S, Rey RA (2006). Time course of the serum gonadotropin surge, inhibins, and anti-Müllerian hormone in normal newborn males during the first month of life. J Clin Endocrinol Metab.

[45] Bergadá I, Bergadá C, Campo S (2001). Role of inhibins in childhood and puberty. J Pediatr Endocrinol Metab.

[46] Andersson AM, Toppari J, Haavisto AM, Petersen JH, Simell T, Simell O, Skakkebaek NE (1998). Longitudinal reproductive hormone profiles in infants: peak of inhibin B levels in infant boys exceeds levels in adult men. J Clin Endocrinol Metab.

[47] Resende EA, Lara BH, Reis JD, Ferreira BP, Pereira GA, Borges MF (2007). Assessment of basal and gonadotropin-releasing hormonestimulated gonadotropins by immunochemiluminometric and immunofluorometric assays in normal children. J Clin Endocrinol Metab.

[48] van Tijn DA, Schroor EJ, Delemarre-van de Waal HA, de Vijlder JJ, Vulsma T (2007). Early assessment of hypothalamic-pituitary-gonadal function in patients with congenital hypothyroidism of central origin. J Clin Endocrinol Metab.

[49] Segal TY, Mehta A, Anazodo A, Hindmarsh PC, Dattani MT (2009). Role of gonadotropin-releasing hormone and human chorionic gonadotropin stimulation tests in differentiating patients with hypogonadotropic hypogonadism from those with constitutional delay of growth and puberty. J Clin Endocrinol Metab.

[50] Bin-Abbas B, Conte FA, Grumbach MM, Kaplan SL (1999). Congenital hypogonadotropic hypogonadism and micropenis: effect of testosterone treatment on adult penile size why sex reversal is not indicated. J Pediatr.

[51] Ogilvy-Stuart AL (2003). Growth hormone deficiency (GHD) from birth to 2 years of age: diagnostic specifics of GHD during the early phase of life. Horm Res.

[52] Ogilvy-Stuart AL, Hands SJ, Adcock CJ, Holly JM, Matthews DR, Mohamed-Ali V, Yudkin JS, Wilkinson AR, Dunger DB (1998). Insulin, insulinlike growth factor I(IGF-I), IGF-binding protein-1, growth hormone, and feeding in the newborn. J Clin Endocrinol Metab.

[53] Kurtoğlu S, Kondolot M, Mazicioğlu MM, Hatipoğlu N, Akin MA, Akyildiz B (2010). Growth hormone, insulin like growth factor-1, and insulinlike growth factor-binding protein-3 levels in the neonatal period: a preliminary study. J Pediatr Endocrinol Metab.

[54] Binder G, Weidenkeller M, Blumenstock G, Langkamp M, Weber K, Franz AR (2010). Rational approach to the diagnosis of severe growth hormone deficiency in the newborn. J Clin Endocrinol Metab.

[55] Binder G, Hettmann S, Weber K, Kohlmüller D, Schweizer R (2011). Analysis of the GH content within archived dried blood spots of newborn screening cards from children diagnosed with growth hormone deficiency after the neonatal period. Growth Horm IGF Res.

[56] Hawkes CP, Grimberg A (2013). Measuring growth hormone and insulin-like growth factor-I in infants: what is normal?. Pediatr Endocrinol Rev.

[57] Hussain K, Hindmarsh P, Aynsley-Green A (2003). Spontaneous hypoglycemia in childhoodis accompanied by paradoxically low serum growth hormone and appropriate cortisolcounterregulatory hormonal responses. J Clin Endocrinol Metab.

[58] Crofton PM, Midgley PC (2004). Cortisol and growth hormone responses to spontaneous hypoglycaemia in infants and children. Arch Dis Child.

[59] Chung ST, Chi CH, Haymond MW, Jeha GS (2015). Infantile Growth Hormone Deficiency and X- Linked Adrenal Hypoplasia Congenita. Jacobs J Pediatr.

[60] Aitkenhead H, Heales SJ (2013). Establishment of paediatric age-related reference intervals for serum prolactin to aid in the diagnosis of neurometabolic conditions affecting dopamine metabolism. Ann Clin Biochem.

[61] Ben-Jonathan N, Hnasko R (2001). Dopamine as a prolactin (PRL) inhibitor. Endocr Rev.

[62] Cheetham T, Baylis PH (2002). Diabetes insipidus in children: pathophysiology, diagnosis and management. Paediatr Drugs.

[63] Saborio P, Tipton GA, Chan JC (2000). Diabetes insipidus. Pediatr Rev.

[64] Wang LC, Cohen ME, Duffner PK (1994). Etiologies of central diabetes insipidus in children. Pediatr Neurol.

[65] Ferlin ML, Sales DS, Celini FP, Martinelli Junior CE (2015). Central diabetes insipidus: alert for dehydration in very low birth weight infants during the neonatal period. A case report. Sao Paulo Med J.

[66] Garel C, Léger J (2007). Contribution of magnetic resonance imaging in nontumoral hypopituitarism in children. Horm Res.

[67] Kitamura E, Miki Y, Kawai M, Itoh H, Yura S, Mori N, Sugimura K, Togashi K (2008). T1signal intensity and height of the anterior pituitary in neonates: correlation with postnatal time. AJNR Am J Neuroradiol.

[68] Dietrich RB, Lis LE, Greensite FS, Pitt D (1995). Normal MR appearance of the pituitary gland in the first 2 years of life. AJNR Am J Neuroradiol.

[69] Argyropoulou M, Perignon F, Brunelle F, Brauner R, Rappaport R (1991). Height ofnormal pituitary gland as a function of age evaluated by magnetic resonanceimaging in children. Pediatr Radiol.

[70] Sari S, Sari E, Akgun V, Ozcan E, Ince S, Saldir M, Babacan O, Acikel C, Basbozkurt G, Ozenc S, Yesilkaya S, Kilic C, Kara K, Vurucu S, Kocaoglu M, Yesilkaya E (2014). Measures of pituitary gland and stalk: from neonate to adolescence. J Pediatr Endocrinol Metab.

[71] Persani L (2012). Clinical review: Central hypothyroidism: pathogenic, diagnostic, and therapeutic challenges. J Clin Endocrinol Metab.

[72] Yagasaki H, Kobayashi K, Nemoto A, Naito A, Sugita K, Ohyama K (2010). Late-onset circulatory dysfunction after thyroid hormone treatment in an extremely low birth weight infant. J Pediatr Endocrinol Metab.

[73] Heckmann M, Hartmann MF, Kampschulte B, Gack H, Bödeker RH, Gortner L, Wudy SA (2005). Assessing cortisol production in preterm infants: do not dispose of the nappies. Pediatr Res.

[74] Higuchi A, Hasegawa Y (2006). Dose Adjustments of Hydrocortisone and L-thyroxine in Hypopituitarism Associated with Cholestasis. Clin Pediatr Endocrinol.

[75] Bouvattier C, Maione L, Bouligand J, Dodé C, Guiochon-Mantel A, Young J (2011). Neonatal gonadotropin therapy in male congenital hypogonadotropic hypogonadism. Nat Rev Endocrinol.

[76] Main KM, Schmidt IM, Toppari J, Skakkebaek NE (2002). Early postnatal treatment of hypogonadotropic hypogonadism with recombinant human FSH and LH. Eur J Endocrinol.

[77] Guthrie RD, Smith DW, Graham CB (1973). Testosterone treatment for micropenis during early childhood. J Pediatr.

[78] Di Iorgi N, Napoli F, Allegri AE, Olivieri I, Bertelli E, Gallizia A, Rossi A, Maghnie M (2012). Diabetes insipidus--diagnosis and management. Horm Res Paediatr.

[79] Van der Kaay DC, Van Heel WJ, Dudink J, van den Akker EL (2014). Transient diabetes insipidus in a preterm neonate and the challenge of desmopressin dosing. J Pediatr Endocrinol Metab.

[80] Korkmaz HA, Demir K, Kılıç FK, Terek D, Arslanoğlu S, Dizdarer C, Ozkan B (2014). Management of central diabetes insipidus with oral desmopressin lyophilisate in infants. J Pediatr Endocrinol Metab.

[81] Stick SM, Betts PR (1987). Oral desmopressin in neonatal diabetes insipidus. Arch Dis Child.

